# TTF-1 Action on the Transcriptional Regulation of Cyclooxygenase-2 Gene in the Rat Brain

**DOI:** 10.1371/journal.pone.0028959

**Published:** 2011-12-13

**Authors:** Chang Ho Yun, Jae Geun Kim, Byong Seo Park, Hye Myeong Lee, Dong Hee Kim, Eun Ok Kim, Joong Jean Park, Jeong Woo Park, Giuseppe Damante, Young Il Kim, Byung Ju Lee

**Affiliations:** 1 Department of Biological Sciences, College of Natural Sciences, University of Ulsan, Ulsan, South Korea; 2 Department of Physiology, College of Medicine, Korea University, Seoul, South Korea; 3 Department of Biomedical Sciences and Technologies, University of Udine, Udine, Italy; 4 Department of Internal Medicine, Ulsan University Hospital, Ulsan, South Korea; University of Hong Kong, Hong Kong

## Abstract

We have recently found that thyroid transcription factor-1 (TTF-1), a homeodomain-containing transcription factor, is postnatally expressed in discrete areas of the hypothalamus and closely involved in neuroendocrine functions. We now report that transcription of cyclooxygenase-2 (COX-2), the rate limiting enzyme in prostaglandin biosynthesis, was inhibited by TTF-1. Double immunohistochemistry demonstrated that TTF-1 was expressed in the astrocytes and endothelial cells of blood vessel in the hypothalamus. Promoter assays and electrophoretic mobility shift assays showed that TTF-1 inhibited COX-2 transcription by binding to specific binding domains in the COX-2 promoter. Furthermore, blocking TTF-1 synthesis by intracerebroventricular injection of an antisense oligomer induced an increase of COX-2 synthesis in non-neuronal cells of the rat hypothalamus, and resulted in animals' hyperthermia. These results suggest that TTF-1 is physiologically involved in the control of thermogenesis by regulating COX-2 transcription in the brain.

## Introduction

Thermoregulation is an essential component of the homeostatic system to maintain body temperature during the febrile response to infection and the challenge of low environmental temperature [1]. Fever is a brain-regulated sickness response, which is triggered by several peripheral signals, and mainly regulated by the hypothalamus [2]. Circulating pyrogen-induced fever is mediated by pro-inflammatory cytokines, such as tumor necrosis factor-α (TNF-α), interleukin 1-β (IL 1-β) and IL-6 [3].

Prostaglandin E2 (PGE2) or its synthesis by action of cyclooxygenase (COX), a rate limiting enzyme for PG synthesis, was shown to play a critical role in the fever response [4], [5]. Two isoforms of COX were found in the early 1990s, and named COX-1 and COX-2 [6]. COX-1 and COX-2 show a high sequence homology, but their catalytic activities and expression patterns are markedly different [7], [8]. COX-1 is expressed constitutively in various cells and tissues [9], while COX-2 expression is inducible in response to growth factors, tumor inducers, hormones, and various inflammatory agents in many cell types [10]. Fever invoked by various inflammatory inducers was suppressed by COX-2-specific inhibitors or by disruption of the COX-2 gene [4], [11], [12], and was strongly associated with COX-2 expression in the brain endothelial cells [13].

In accordance with the notion of inducible expression of COX-2, its promoter region contains a number of binding domains for transcription factors [14], most of which are stimulatory. In the brain, neuronal COX-2 is constitutive, while its expression in non-neuronal cells is stimulated by pyrogen through the action of nuclear factor kappa B [15], [16]. In this study we have identified a specific action of thyroid transcription factor-1 (TTF-1) for the inhibition of COX-2 transcription in the rat hypothalamus and its physiological role for fever control.

Though TTF-1 was first identified in the thyroid gland [17], it was also reported as Nkx2.1 (a member of the Nkx family of homeobox genes) essential for normal development of embryonic diencephalon [18]. TTF-1 expression persists after birth in defined glial and neuronal subsets of the forebrain [19]. Specifically, TTF-1 was detected in some neuronal cells of the rat hypothalamus such as neurons expressing gonadotropin releasing hormone (GnRH) [19], pituitary adenylate cyclase-activating polypeptide (PACAP) [20], and proopiomelanocortin (POMC) and agouti-related peptide (AgRP) [21]. In addition to its neuronal expression, TTF-1 was also expressed in non-neuronal cells in the postnatal rat brain, such as ependymoglial cells of the third ventricle and median eminence [19], in the tanycytes and/or astrocytic tanycytes of the subfornical organ [22], and in the epithelial cells of the choroid plexus [23].

We now report that TTF-1 binds to its binding domains in COX-2 promoter and inhibits COX-2 transcription in non-neuronal cells in the brain. In addition, obstruction of TTF-1 synthesis stimulated COX-2 expression in the hypothalamic non-neuronal cells and resulted in an increase in body temperature.

## Results

### Expression of TTF-1 in the non-neuronal cells of preoptic area (POA)

**Figure 1:**
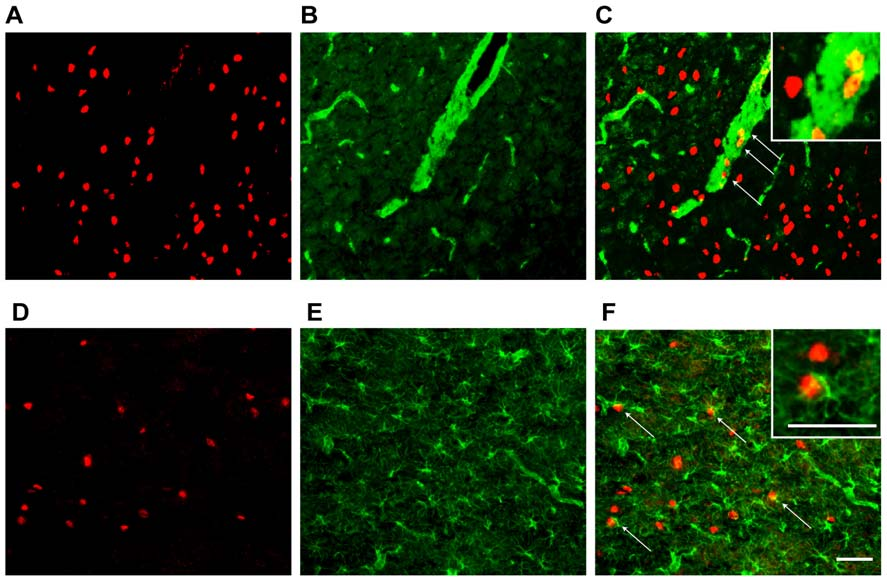
Expression of TTF-1 in non-neuronal cells of the POA. Double IHC was performed on brain sections derived from 2-month-old male rats. TTF-1 protein (red, *A, C, D* and *F*) was detected with a monoclonal antibody. Endothelial cells of blood vessels (green, *B* and *C*) were identified using antibody against vWF, an endothelial cell-specific marker. Astroglial cells (green, *E* and *F*) were determined using GFAP antibody. *A*, red fluorescence signals representing TTF-1 immuno-positive cells in the POA. *B*, green signals revealing vWF-ir in the endothelial cells of blood vessel on the same section with *A*. *C*, merged image of *A* and *B* showing some cells co-expressing TTF-1 and vWF (indicated as arrows). Inset highlighting higher magnification image of TTF-1 colocalized with vWF. *D* and *E*, TTF-1 (red, *D*) and GFAP (green, *E*) immunoreactivities in cells of the POA. *F*, merged image of *D* and *E* revealing some cells with colocalization of TTF-1 and GFAP (arrow). Higher magnification image in inset highlighting colocalization of TTF-1 and GFAP. Scale bar = 50 µm.

Previously, our reports have shown that TTF-1 is expressed in some non-neuronal cells in the rat brain, such as ependymoglial cells, tanycytes and epithelial cells of the third ventricle, subfornical organ and choroid plexus, respectively [19], [22], [23]. Therefore, in this study we tried to identify TTF-1 expression in the COX-2 expressing non-neuronal cells. Because the endothelial cells of blood vessel and astrocytes in the POA are important sites of the formation of PGs by COX-2 action [13], [24], we performed double immunohistochemistry (IHC) using a TTF-1 antibody and an antibody against von Willebrand factor (vWF), a marker for endothelial cells of blood vessel or an antibody for glial fibrillary acidic protein (GFAP), a marker for astrocytes, on the brain sections from male rats. As shown in Fig. 1, TTF-1-immunoreactivity (ir) (Fig. 1A and D) is widely distributed in the cells throughout the POA. Some cells showing TTF-1-ir also express vWF-ir (Fig. 1B and C) or GFAP-ir (Fig. 1E and F), suggesting that TTF-1 is expressed in some non-neuronal cells in the POA, such as endothelial cells of blood vessel and astrocytes. A similar cellular co-localization of TTF-1 and vWF or GFAP was observed in other brain regions examined (data not shown).

### Effect of intracerebroventricular (icv) injection of antisense (AS) TTF-1 oligodeoxynucleotide (ODN) on COX-2 expression in the hypothalamus

**Figure 2:**
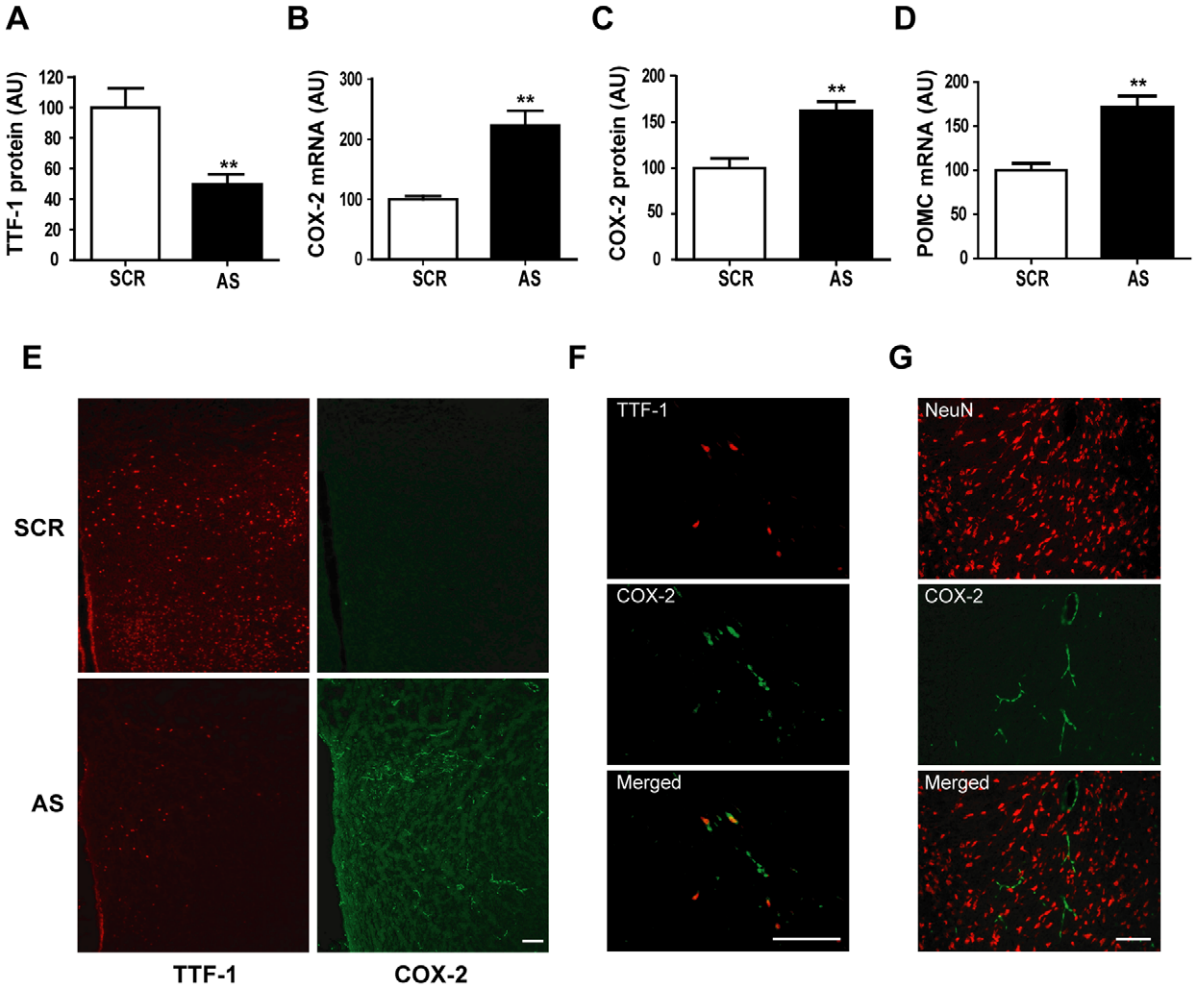
Effect of TTF-1 synthesis blockade on the COX-2 expression in the hypothalamus. The AS TTF-1 ODN or SCR ODN was injected into the lateral ventricle of 2-month-old male rats. One day after the injection, the hypothalamus was collected for western blot and real-time PCR analyses, or was examined by IHC. *A*, western blots showing a decrease in TTF-1 protein level caused by administration of the AS TTF-1 ODN (AS) compared with SCR ODN. *B and C*, AS TTF-1 ODN significantly increased both COX-2 mRNA (*B*) and protein (*C*) levels determined by real-time PCR analysis and western blotting. *D*, real-time PCR analysis showing increased POMC mRNA by the AS ODN (n = 6). **, *p*<0.01 *versus* SCR. *E*, the POA section showing a decrease of TTF-1-ir (red) and an increase of COX-2-ir (green) caused by the AS TTF-1 ODN. *F,* higher magnification images showing co-localization of TTF-1-ir (red) and COX-2-ir (green) in the AS ODN injected rat POA. Note that COX-2-ir appears in some cells expressing TTF-1-ir (merged, arrow). *G*, higher magnification images showing COX-2-ir (green) in the AS ODN injected rat POA. Notice that COX-2 is present only in cells with an absence of the NeuN-ir (red). Scale bar = 100 µm.

To determine whether *in vivo* inhibition of TTF-1 synthesis affects COX-2 synthesis in the hypothalamus, we injected a well-defined blocking system of TTF-1 synthesis [21], [22], [23], AS TTF-1 ODN, or its SCR (scrambled) sequence into the lateral ventricle of male rats. One day after the injection, the hypothalamus was collected for measurement of TTF-1 and COX-2 expression levels. As shown in Fig. 2A, the AS TTF-1 ODN injection effectively decreased the content of TTF-1 protein in the hypothalamus, as determined by western blot analysis. This reduction in TTF-1 protein level was accompanied by an induction in COX-2 mRNA and protein in the hypothalamus (Fig. 2B and C). POMC mRNA, negatively regulated by TTF-1 [21], was also increased by the AS TTF-1 ODN (Fig. 2D), further indicating that the AS ODN is effective.

IHC results confirmed that the AS TTF-1 ODN clearly decreased TTF-1-ir in the POA section (Fig. 2E, left), which resulted in a dramatic increase of COX-2-ir (Fig. 2E, right). COX-2-ir was rarely or never observed in the SCR ODN-injected section, suggesting that its expression is strongly suppressed by TTF-1 in this condition. Thus, we tried to colocalize COX-2 and TTF-1 in the AS TTF-1 ODN injected rat POA. Though TTF-1-ir was decreased by the AS TTF-1 ODN (as shown in Fig. 2C, left), some cells positive to TTF-1-ir was also positive to the COX-2-ir in the AS TTF-1 ODN injected condition (Fig. 2F), while no double positive cell was observed in the SCR ODN injected animals (data not shown). The AS TTF-1 ODN induced COX-2-ir was localized in non-neuronal cells negative for neuron specific nuclear protein (NeuN)-ir in the POA (Fig. 2G); whereas it showed specific distribution in the cells located in blood vessel-like structures (as identified by vWF in Fig. 1B and C). These data suggest that blockade of TTF-1 synthesis induces non-neuronal COX-2 expression in the endothelial cells of blood vessels in the hypothalamus where is an important brain structure for the fever response [13].

In agreement with a previous study [25], constitutive neuronal COX-2-ir was observed in several brain regions, such as the cerebral cortex, hippocampus and piriform cortex in both SCR ODN and AS TTF-1 ODN treated animals, but no change to COX-2-ir was found in these neuronal regions between the treatments (Fig. S1). Interestingly, the AS ODN induced COX-2 expression only in the blood vessel cell-like structures (Fig. S1D, E, G, and H), while COX-2-ir was absent in these structures of SCR ODN-injected control (Fig. S1A and B). This might be due to these cells expressing inducible non-neuronal COX-2 [26] which is under inhibitory control of TTF-1. Though we were unable to detect TTF-1-ir in these regions, a previous study reported TTF-1 expression in the cerebral cortex and hippocampus of the mouse brain [27].

### TTF-1 inhibits COX-2 transcription in non-neuronal cells

**Figure 3:**
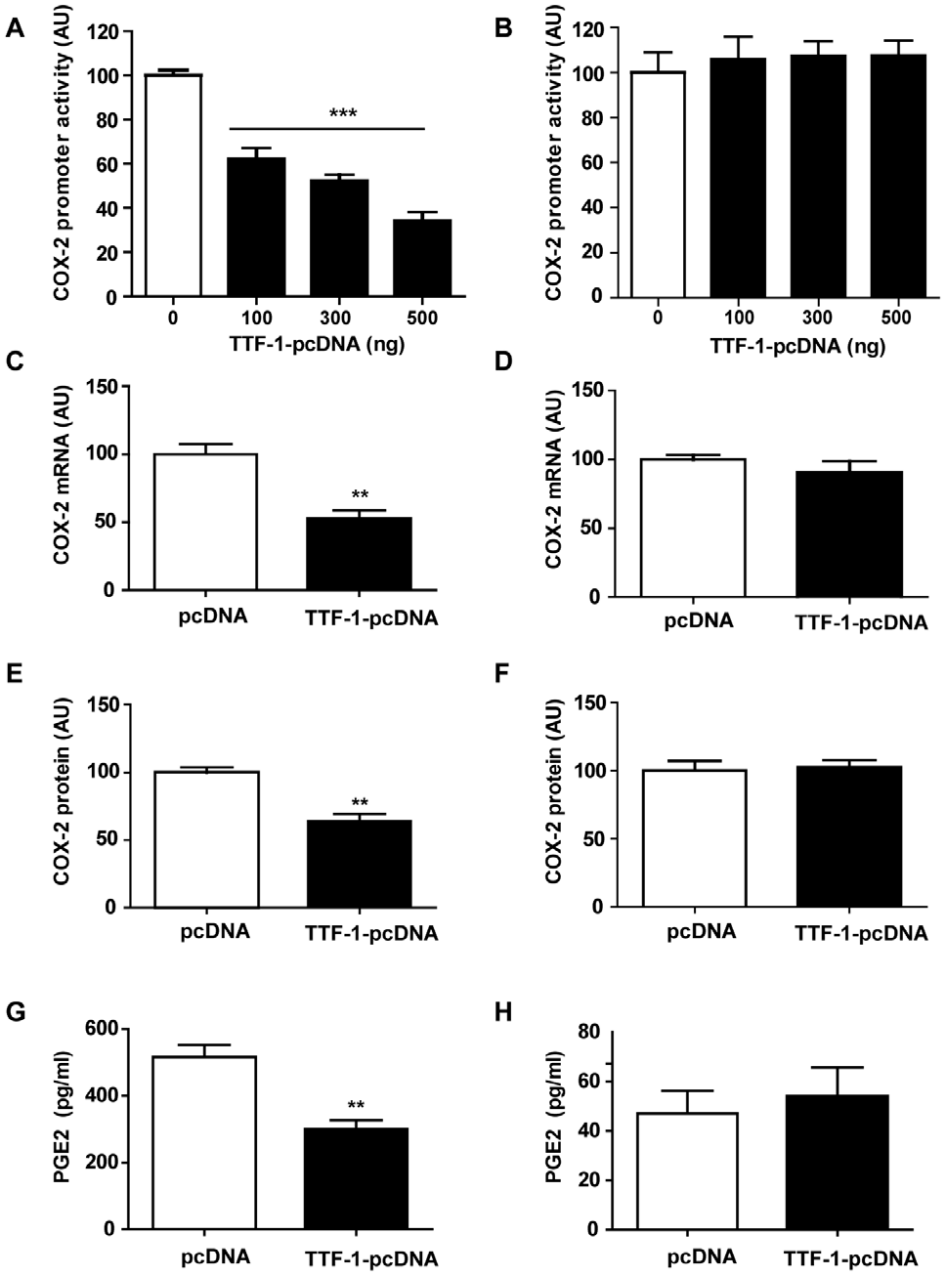
Regulation of COX-2 transcription by TTF-1. Luciferase reporter constructs (pGL2) containing 5′-flanking region of the rat COX-2 gene were cotransfected into C6 glioma and B35 neuroblastoma cells with an expression vector carrying the rat TTF-1-coding region (TTF-1-pcDNA) at the final concentrations indicated. The cells were harvested for luciferase and β-galactosidase assays 24 h after transfection. *A*, dose-dependent inhibition of the COX-2 promoter activity by increasing concentrations of TTF-1 in C6 cells. Each value represents mean±SEM (*n* = 6). ***, *p*<0.001 *versus* 0 ng. *B*, absence of TTF-1 effect on COX-2 promoter in B35 cells. *C and D,* data from real-time PCR analysis showing effect of TTF-1 (TTF-1-pcDNA, 500 ng) on the endogenous COX-2 mRNA expression in the C6 cells (*C*) and B35 cells (*D*). *E and F*, data from western blotting revealing effect of TTF-1 on the COX-2 protein in the C6 cells (*E*) and B35 cells (*F*). *G and H*, PGE2 release from C6 cells (*G*) and B35 cells (*H*) by overexpression of TTF-1. Each value represents mean±SEM (*n* = 6). **, *p*<0.01 *versus* pcDNA.

To confirm the inhibitory effect of TTF-1 on the transcription of the COX-2 gene, we performed promoter assays using a luciferase construct containing the rat COX-2 promoter region (-2698 to +32) and a rat TTF-1 expression plasmid (TTF-1-pcDNA) in C6 glioma and B35 neuroblastoma cells. COX-2 promoter activity was gradually decreased by addition of TTF-1 expression vector in a dose-dependent manner in C6 cells (Fig. 3A), but did not show any change in the B35 cells (Fig. 3B). To further confirm effect of TTF-1 on the endogenous COX-2 expression in the C6 and B35 cells, we performed real-time PCR and western blot analysis of samples extracted from the cells transfected with TTF-1 expression vector. Overexpression of TTF-1 decreased mRNA and protein levels of COX-2 in the C6 glioma cells (Fig. 3C and E), but not in the B35 neuroblastoma cells (Fig. 3D and F). In accordance with COX-2 expression, PGE2 release was also decreased by TTF-1 only in the C6 cells (Fig. 3G and H).

Consistent with *in vivo* results shown above, results from promoter assays, real-time PCR, and western blot analysis suggest that TTF-1 exerts an inhibitory effect on COX-2 transcription only in non-neuronal cells.

### TTF-1 directly binds to its binding sites in the COX-2 promoter

**Figure 4:**
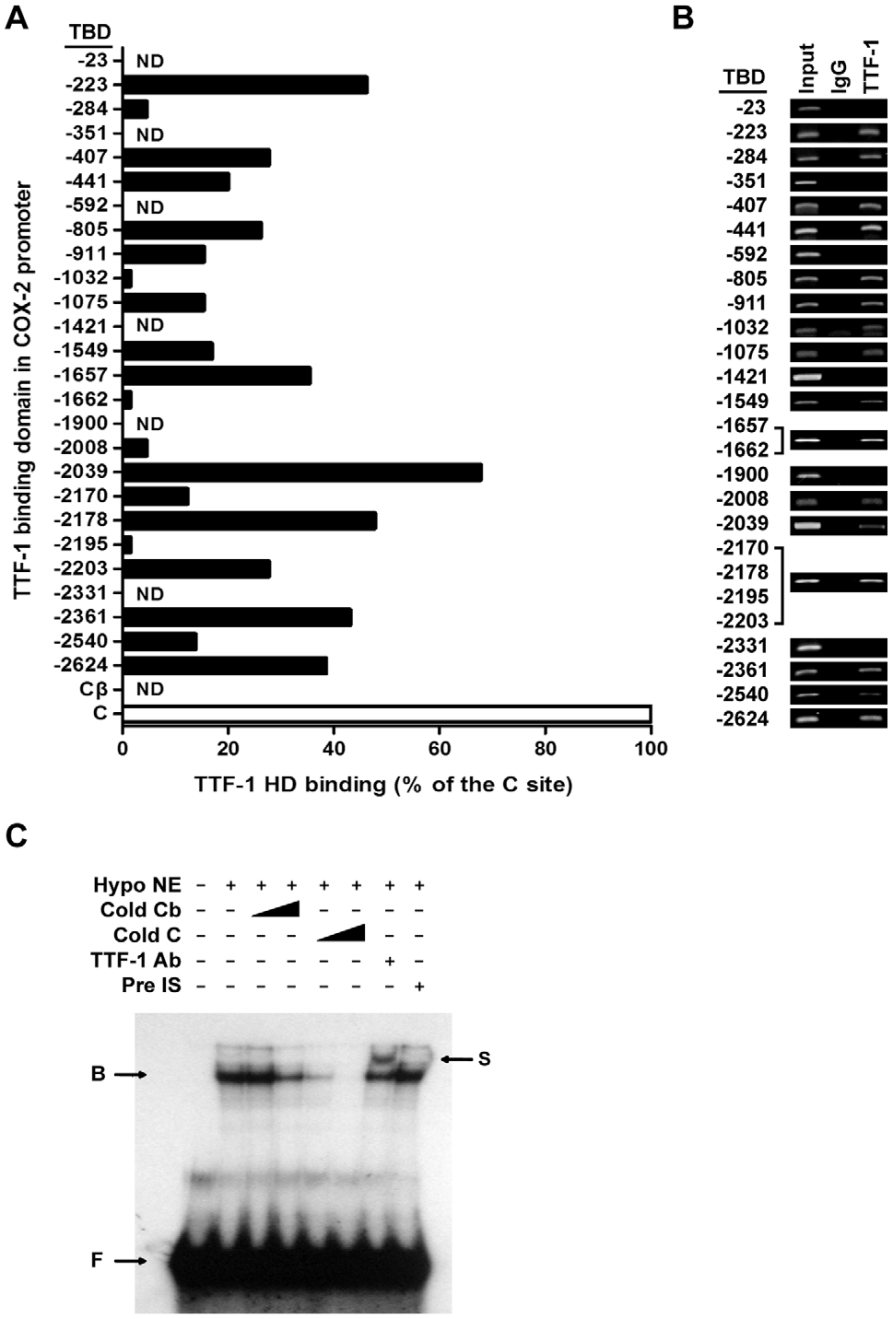
EMSAs and ChIP assays. EMSAs were performed with double-stranded oligomer probes containing the putative TTF-1 binding sites shown in Fig. S2 and table 1. *A*, relative binding activities calculated as a percentage of TTF-1 HD binding to the positive control probe C. Cβ, negative control probe carrying mutations in the TTF-1 binding domain (TBD). ND, not detectable. *B,* ChIP assays of rat COX-2 promoter DNA using TTF-1 Ab. DNA was immunoprecipitated from C6 cells with TTF-1 Ab (TTF-1) or IgG (as a negative control), and was PCR-amplified with primer sets, shown in Information S1, for COX-2 promoter fragments including TTF-1 binding sites indicated as numbers at TBD. Input represents the DNA extracted from the C6 cells before immunoprecipitation. *C*, hypothalamic nuclear extracts were incubated with oligonucleotide probes containing −2039 TTF-1 binding site, in the presence (+) or absence (-) of 5- or 20-fold excess of cold oligonecleotide C and Cβ and TTF-1 antibody (TTF-1 Ab) or preimmune serum (Pre IS). Incubation of nuclear proteins with a TTF-1 Ab prior to the protein-DNA binding reaction delays (arrow S, supershift) the migration of the protein-DNA complex (arrow B). F, free probe.

Electrophoretic mobility shift assays (EMSAs) were performed to determine the ability of synthetic TTF-1 homeodomain (HD) to recognize the putative TTF-1 binding sites present in the COX-2 promoter. Double-stranded ODN probes, containing the presumptive TTF-1 binding sites (Fig. S2) and their flanking sequences, shown in Table 1, were employed. Of the 26 putative binding sites, 20 were recognized by TTF-1 HD (Fig. 4A). The site at −2039 showed the strongest signal that reached about 70% of the positive control probe C. The sites at −2624, −2361, −2203, −2178, −1657, −805, −407 and −223 revealed moderate binding activity at least about 25% of probe C. Other putative binding domains showed only very low or no binding activity.

**Table 1:** EMSA oligonucleotide probes.

Location in the promoter region of COX-2 gene	Probe sequences
C*	5′-CACTGCCCAT**CAAG**TGTTCTTGA-3′
**Cβ** ^**+**^	5′-CACTGCCCAGTCACGCGTTCTTGA-3′
**−2624**	5′- GGAAATT**CAAG**CAGCAGA-3′
**−2540**	5′- AGCTTCT**CAAG**GAAACTT-3′
**−2361**	5′- GAACCAT**CTTG**ATTTAGT-3′
**−2331**	5′- ATATTCC**CTTG**TCATCAG-3′
**−2203**	5′- AATTGCT**CTTG**TCCTCAA-3′
**−2195**	5′- TTGTCCT**CAAG**GTCTAAG-3′
**−2178**	5′- GTTTCTT**CTTG**AGTTCTT-3′
**−2170**	5′- TTGAGTT**CTTG**TGTAACT-3′
**−2039**	5′- TAAATTT**CAAG**GAGTCTG-3′
**−2008**	5′- CCTATGC**CTTG**CTTTTCC-3′
**−1900**	5′- CCTGAGA**CTTG**CTCTGTA-3′
**−1662**	5′- TGAGTAC**CTTG**ACAAGAG-3′
**−1657**	5′- ACCTTGA**CAAG**AGTGTGG-3′
**−1549**	5′- CAGAAAA**CAAG**AACTACT-3′
**−1421**	5′- AAAGGGC**CTTG**GTGACAT-3′
**−1075**	5′- TTCATGC**CAAG**AACGTAC-3′
**−1032**	5′- TCATTTT**CTTG**TTTTACT-3′
**−911**	5′- TTATTAT**CAAG**CAATGTT-3′
**−805**	5′- ATATCTT**CTTG**TAAACGT-3′
**−592**	5′- CCTGGGG**CTTG**CTAGGAC-3′
**−441**	5′- AGAGCAG**CAAG**CACGTCA-3′
**−407**	5′- GGAGAGG**CAAG**GGGATTC-3′
**−351**	5′- ACATTCT**CTTG**CTCCTCC-3′
**−284**	5′- AGCCAGT**CTTG**GAGCAGG-3′
**−223**	5′- CAGCTCT**CTTG**GCACCAC-3′
**−23**	5′- TTAAAAG**CAAG**GTTCTCC-3′

Each sequence represents the sense strand of probes; core TTF-1 binding motifs are underlined.

*and + denote positive and negative control probes, respectively (see text).

To further determine *in vivo* interactions of TTF-1 with its binding domains in the COX-2 promoter, chromatin immunoprecipitation (ChIP) assays were performed using a TTF-1 antibody. The precipitated DNA was amplified using PCR primer sets specific to the TTF-1 binding site in the COX-2 promoter. Interestingly, primer sets targeted to the positive TTF-1 binding domains observed in EMSA (Fig. 4A) amplified fragments encompassing each TTF-1 binding motif (TBD) in the COX-2 promoter region (Fig. 4B). However, PCR amplification, using primer sets for the domains revealing no binding with TTF-1 HD (Fig. 4A), did not generate any specific band (Fig. 4B).

To test whether the rat hypothalamic TTF-1 is able to specifically interact with the COX-2 promoter, EMSAs were performed with nuclear extracts from the rat hypothalamus, using probe -2039 which showed the strongest binding activity (Fig. 4A). Nuclear proteins from the rat hypothalamus strongly bound to the −2039 ODN probe (Fig. 4B). The interaction of the labeled probe with hypothalamic nuclear proteins was reduced by addition of a 5- or 20-fold excess of unlabeled ODN C. In contrast, an ODN probe carrying a mutated core TTF-1 binding sequence C (Cβ) unable to bind TTF-1 was ineffective. Pre-incubation of hypothalamic nuclear proteins with a TTF-1 antibody delayed the migration of the protein-DNA complex, indicating that TTF-1 was indeed part of this complex.

### Essential TTF-1 binding sites for the transcriptional inhibition of COX-2 gene

**Figure 5:**
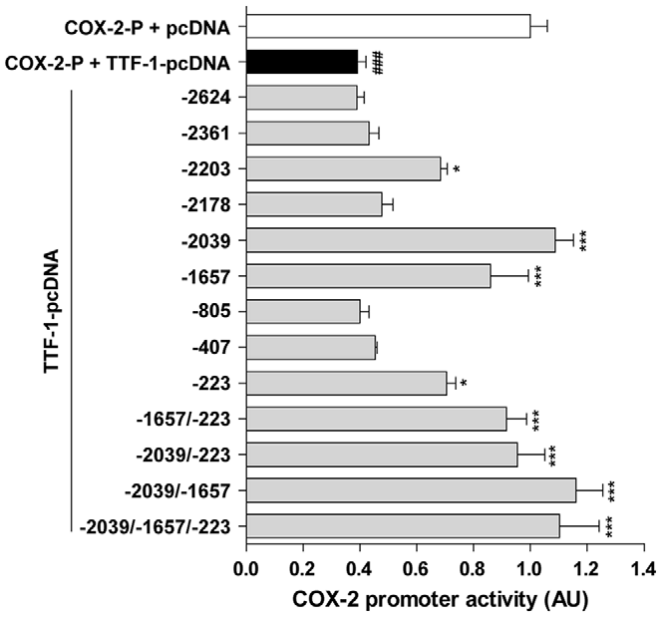
Effect of site-specific deletion of TTF-1 binding core motifs on the TTF-1-induced inhibition of COX-2 promoter activity. TTF-1 expression vector (TTF-1-pcDNA, 500 ng) was cotransfected with nine single mutants of the COX-2 promoter (COX-2-P) deleted with core TTF-1 binding sites (showing relatively strong or moderate binding with TTF-1 in EMSAs) or with combined mutants deleted with −2039, −1657, and/or −223 sites. Positions of the deleted binding sites are indicated. The data are the means±SEM (*n* = 4). ###, *p*<0.001 *versus* COX-2-P + pcDNA; *, *p*<0.05; ***, *p*<0.001 *versus* COX-2-P + TTF-1-pcDNA.

To determine whether the TTF-1 binding sites recognized by EMSAs are functionally active, we selected 9 TTF-1 binding motifs showing medium or strong binding activity in the EMSAs (Fig. 4A) and deleted each of 5′-CAAG-3′ (or 5′-CTTG-3′) core motifs from the binding domains, and examined the ability of TTF-1 to inhibit the mutated COX-2 promoters in C6 cells (Fig. 5). Deletion of the −2039 binding site (showing the strongest binding activity) completely abolished the inhibitory effect of TTF-1 on the COX-2 promoter activity, suggesting that the site at −2039 is the most important site for the regulation of COX-2 transcription by TTF-1. As shown in Fig. 5, deletion of some sites (−2203, −1657 and −223) resulted in a partial reversion (about 70% to 85% of the control COX-2 promoter activity without TTF-1) from the TTF-1-induced inhibition of the COX-2 promoter activity (40% of the control COX-2 promoter activity without TTF-1). Combined deletions of the binding motifs at −2039, −1657 and/or −223 sites resulted in a complete disappearance of inhibitory TTF-1 action on the COX-2 promoter activity. Combined deletions including both −2039 and −1657 sites looked a little bit more effective than other combinations. However, no significant change was observed when other sites were deleted, suggesting that only part of the TTF-1 binding domains identified by EMSAs are functionally active. Results from promoter assays demonstrate that TTF-1 is a functionally active inhibitor of COX-2 transcription by binding to its binding domains in the COX-2 promoter region.

### Effect of TTF-1 synthesis blockade on the regulation of body temperature

**Figure 6:**
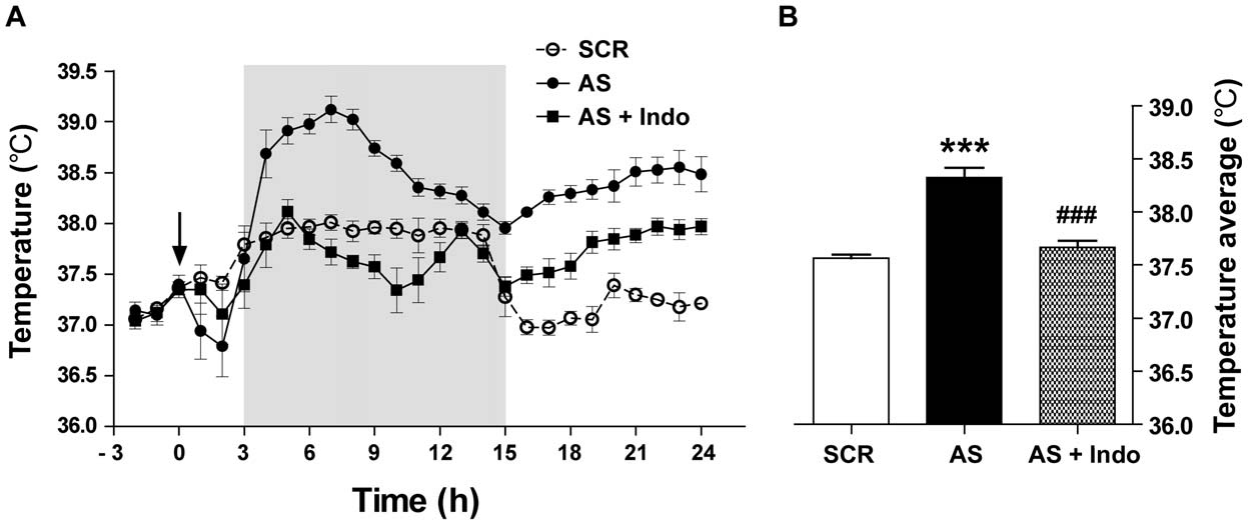
Effect of TTF-1 synthesis blockade by AS TTF-1 ODN on the change of body temperature. Body temperature was measured for 24 h after icv administration of AS TTF-1 ODN or SCR ODN. *A*, temperature began to increase 2 h after icv injection (arrow at 0 h) of the AS TTF-1 ODN (AS) and remained high until about 20 h after the injection compared with SCR ODN injected group (SCR). To determine involvement of prostaglandins in the AS-induced hyperthermia, rats were i.p.-injected with indomethacin (Indo) 30 min prior to the injection of the AS. Pretreatment of Indo significantly reduced the AS-induced increase of body temperature. The shaded area indicates dark period. The values represent means±SEM (*n* = 6). *B*, mean temperature after the injection indicating a significant difference between groups. ***, p<0.001 versus SCR; ###, p<0.001 versus AS.

To determine if blocking TTF-1 synthesis in the brain exerts a physiological change in whole body levels, we evaluated the body temperature of rats icv injected with the AS TTF-1 ODN. A blockade of TTF-1 synthesis by the AS ODN dramatically increased body temperature beginning 2 h after the injection; this increase continued the whole observation period (Fig. 6). To further confirm whether the effect of the AS ODN on the body temperature is caused by change in PG synthesis, rats were i.p. injected with indomethacin, a COX inhibitor, 30 min prior to the injection of AS TTF-1 ODN, and their body temperature was observed. Indomethacin significantly mitigated the AS TTF-1 ODN-induced hyperthermia during the observation period (Fig. 6), suggesting that the blockade of TTF-1 synthesis by the AS ODN induces a fever response via decreasing the inhibitory action of TTF-1 on COX-2 expression.

## Discussion

This study demonstrates the inhibitory action of TTF-1 in the control of inducible non-neuronal COX-2 in the brain, and its effect on COX-2-induced fever. Our histological data revealed that TTF-1 is expressed in some portion of the non-neuronal cells, such as astrocytes and endothelial cells of blood vessels, the main sites of inducible COX-2 expression in the POA. Promoter assays showed that TTF-1 inhibits COX-2 transcription in the glial cells but not in the neuroblastoma cells. In agreement with these *in vitro* data, inhibition of TTF-1 synthesis resulted in an increased expression of non-neuronal COX-2 in the hypothalamus, which caused hyperthermia in the rats.

In earlier studies we have found that TTF-1 targets several downstream genes such as GnRH, proenkephalin, angiotensinogen, aquaporin-1, ErbB-2, PACAP, POMC and AgRP in the neuronal or non-neuronal cells in the rat brain [19], [20], [21], [22], [23]. These genes were identified as TTF-1 targets based on the presence of well conserved TTF-1 binding motifs in their 5′-flanking regions. Surprisingly, the COX-2 promoter has 26 putative TTF-1 binding sites. Among these multiple binding sites, twenty were recognized by binding with the TTF-1 HD: one relatively strong binding site (at −2039) and 8 sites showing moderate binding activity. Four of 9 single deletions within the aforementioned sites resulted in a significant reversion of the TTF-1-dependent decrease of COX-2 promoter activity, suggesting that TTF-1 directly inhibits the COX-2 transcription by binding to these recognition sites. The finding that the COX-2 promoter contains several functional TTF-1 binding sites is not extraordinary. In fact, others and we have already reported the presence of a relatively high number of binding sites for TTF-1 [23], [28] as previously reported for other transcription factors [29], [30].

Interestingly, TTF-1 inhibited COX-2 transcription only in C6 cells, but not in B35 cells. The C6 cell line originated from a chemically induced rat brain tumor and expresses GFAP and vimentin [31], [32], which is a property of undifferentiated astrocytic cell type [33]. B35 cells were derived from a neonatal rat brain tumor displaying neuronal characteristics: showing membrane excitability, expression of enzymes for neurotransmitters and neuron-specific enolase [34]. Thus our results indicate that TTF-1 is a transcriptional repressor specific to non-neuronal COX-2. Consistent with this notion, an *in vivo* blockade of TTF-1 synthesis by AS TTF-1 ODN stimulated COX-2 expression only in non-neuronal cells (likely endothelial cells of blood vessel) but not in neuronal cells.

The brain is one of the few organs where COX-2 is expressed constitutively. COX-2 expressing neurons have been observed in the brain structures like the cortex, hippocampus and amygdale [25]. However, during an immune challenge, non-neuronal cells have been reported to be the main source of PGs through COX-2 action in the brain [13], [35]. Studies using selective COX-2 inhibitors or COX-2-deficient mice suggest a critical role of COX-2 as a mediator of fever induced by lipopolysaccharide (LPS) [4], [35], [36]. Fever response is caused by PGE2 released mainly from endothelial cells of the hypothalamic blood vessels [13]. PGE2 diffuses out of these endothelial cells into thermoregulatory neurons in the POA, the center for body temperature control [37], [38]. Then, EP3 receptors mediate the pyretic action of PGE2; mutant mice lacking this receptor do not develop fever after administration of PGE2, IL-1 or LPS [5].

In this study, we demonstrated that the icv administration of AS TTF-1 ODN not only increased expression of non-neuronal COX-2 in the hypothalamus, but also elevated body temperature. This increase was clearly reversed by pretreatment with a COX inhibitor (indomethacin), indicating that COX-2 (and its end product, PGE2), is involved in the hyperthemia induced by AS TTF-1 ODN.

To inhibit TTF-1 expression *in vivo*, we designed and applied an AS TTF-1 ODN [20], [21], [22], [23] in this study. Although icv administration of the AS ODN may diffuse to areas adjacent to the ventricular region, we demonstrated, using IHC and immunoblot analysis, that injection of the AS ODN into the lateral ventricle induced a marked decrease in TTF-1 availability in the hypothalamus, and thus resulted in related changes in the physiology, such as an increase of non-neuronal COX-2 expression and hyperthermia.

We have recently reported that hypothalamic TTF-1 was down-regulated by administration of leptin, an adipocyte-derived hormone, and was involved in the regulation of feeding behavior via the melanocortin pathway [21]. Although the major function of leptin in the hypothalamus is regulation of appetite, previous studies showed that this cytokine-like peptide can directly regulate inflammation via induction of COX-2 in brain endothelial cells [39], [40], [41]. Another study showed that TNF-α, a major proinflammatory cytokine acting through COX-2, significantly decreased TTF-1 expression as well as formation of TTF-1-DNA complexes in thyroid cells [42]. Therefore, TTF-1 may be an additional novel pathway for the action of the aforementioned proinflammatory cytokines on the regulation of COX-2 synthesis. Further studies are obviously required to test this hypothesis.

In summary, our results show that non-neuronal COX-2 in the hypothalamus is under transcriptional repression by TTF-1, a homeodomain containing transcription factor, and this regulatory mechanism plays an important role in the control of body temperature.

## Materials and Methods

### Animals

Two-month-old male Sprague-Dawley rats (Daehan Animal Breeding Company, Chungwon, Korea) were housed in a room with a conditioned photoperiod (12-h light/12-h darkness, lights on from 6∶00 a.m. to 6∶00 p.m.) and temperature (23–25°C) just after arrival and allowed *ad libitum* access to tap water and pelleted rat chow. Animal experiments were conducted in accordance with the regulations of the University of Ulsan and the National Institutes of Health Guide for the Care and Use of Laboratory Animals. The Institutional Review Board of University of Ulsan approved the experimental procedures (permission number UOU-2010-012).

### DNA constructs

A luciferase reporter plasmid (pGL2; Promega, Madison, WI) containing the rat COX-2 promoter (Fig. S2) (NCBI GenBank database accession No. L11611) was kindly provided by Dr. Ojeda (Oregon National Primate Research Center/Oregon Health and Science University, Beaverton, Oregon). Mutant COX-2 promoter constructs carrying deletions of the TTF-1 binding sites were generated using the QuikChange^TM^ site-directed mutagenesis kit (Stratagene, La Jolla, CA) according to the manufacturer's instructions; the intended mutations were confirmed by sequencing analysis. The oligodeoxynucleotide primers used were (a) a primer set for deletion of −2624 (sense primer, 5′-CAT TGC TGG AAA TTC AGC AGA AGA GGG C-3′; antisense primer, 5′-GCC CTC TTC TGC TGA ATT TCC AGC AAT G-3′), (b) a primer set for deletion of −2361 (sense primer, 5′-GAG AGG TTG AAC CAT ATT TAG TTT GGG AC-3′; antisense primer, 5′-GTC CCA AAC TAA ATA TGG TTC AAC CTC TC-3′), (c) a primer set for deletion of −2203 (sense primer, 5′-CTG CCT TTC AAA ATT GCT TCC TCA AGG TC-3′; antisense primer, 5′-GAC CTT GAG GAA GCA ATT TTG AAA GGC AG-3′), (d) a primer set for deletion of −2178 (sense primer, 5′-GGT CTA AGT TTC TTA GTT CTT GTG TAA CTC-3′; antisense primer, 5′-GAG TTA CAC AAG AAC TAA GAA ACT TAG ACC-3′), (e) a primer set for deletion of -2039 (sense primer, 5′-GTC TTT AAA TTT GAG TCT GAA GG-3′; antisense primer, 5′-CCT TCA GAC TCA AAT TTA AAG AC-3′), (f) a primer set for deletion of −1657 (sense primer, 5′-GAT TTG AGT ACC TTG AAG TGT GGA TTT TTA C-3′; antisense primer, 5′-GTA AAA ATC CAC ACT TCA AGG TAC TCA AAT C-3′), (g) a primer set for deletion of −805 (sense primer, 5′-GCC ATA GCA TAT CTT TAA ACG TAA ACG TGG AC-3′; antisense primer, 5′-GTC CAC GTT TAC GTT TAA AGA TAT GCT ATG GC-3′), (h) a primer set for deletion of −407 (sense primer, 5′-GGG GAG AGG GGG ATT CCC TTA GTT AG-3′; antisense primer, 5′-CTA ACT AAG GGA ATC CCC CTC TCC CC-3), (i) a primer set for deletion of −223 (sense primer, 5′-GGG CGG TGC AGC TCT GCA CCA CTT TGG GC-3′; antisense primer, 5′-GCC CAA AGT GGT GCA GAG CTG CAC CGC CC-3′).

### Real-time PCR

RNA was isolated from the hypothalamus and cell lines using TRI reagent (Sigma-Aldrich, St. Louis, MO). The isolated RNA samples were reverse-transcribed and amplified using real-time PCR with the following primer sets: COX-2 sense primer, 5′-ACC AGA GCA GAG AGA TGA AA-3′; antisense primer, 5′-GAG AGA CTG AAT TGA GGC AG-3′; POMC sense primer, 5′-GCT AGG TAA CAA ACG AAT GG-3′; POMC antisense primer, 5′-GCA TTT TCT GTG CTT TCT CT-3′; glyceraldehydes-3-phosphate dehydrogenase (GAPDH) sense primer, 5′-TGT GAA CGG ATT TGG CCG TA-3′; and antisense primer, 5′-ACT TGC CGT GGG TAG AGT CA-3′. Real-time PCR was carried out in capillaries of the DNA Engine Opticon Continuous Fluorescence Detection System (MJ Research Inc., Waltham, MA) for approximately 40 cycles as follows: at 94 for 30 sec, 56 for 30 sec, and 73 for 35 sec.

### Western blotting

Protein from the hypothalamus was homogenized in T-PER lysis buffer (Pierce Chemical CO., Rockford, IL) containing a protease inhibitor cocktail (1 mM PMSF, 10 µg/ml leupeptin, and 3 mM aprotinin) and 1 mM sodium orthovanadate. Extracted protein (15 µg) was separated by SDS-PAGE and was transferred to a membrane by electrophoretic transfer. The membrane was incubated with mouse anti-TTF-1 antibody (clone 8G7G3/1, NeoMarkers, Fremont, CA) or rabbit anti-COX-2 antibody (Cayman, Ann Arbor, MI). Immunoreactivity was detected with an enhanced chemiluminescence kit (Amersham Biosciences, Little Chalfont, UK).

### Tissue preparation

Rats were deeply anesthetized with tribromoethanol (250 mg/kg body weight; Sigma-Aldrich) and perfused transcardially with 100 ml of 0.1 M phosphate buffer (PB), pH 7.5 followed by 100 ml of 3% paraformaldehyde in 0.1 M PB. The brains were removed and cryoprotected in 0.1 M PB with 20% sucrose overnight at 4°C. The brains were placed into inert mounting medium (OCT compound, Sakura, Torrance, USA) in plastic moulds, transfered to precooled dry ice with ethanol and frozen. Sections were cut 30 µm on a cryostat microtome. Sections were stored at −80°C until ready for use.

### IHC

The brain sections were dried overnight at room temperature. The dried sections were boiled in 10 mM citrate buffer, pH 6.0, for 20 min and allowed to cool to room temperature for 30 min. Sections were then incubated for 30 min at room temperature in a blocking solution containing 3% skim milk and 0.3% Triton X-100 in 0.1 M PB. After incubation, the sections were washed with 0.1 M PB, and incubated with primary antibodies [mouse anti-TTF-1 antibody (1∶400; NeoMarkers), rabbit anti-TTF-1 antibody (1∶1000; Santa Cruz Biotechnology, Santa Cruz, CA), rabbit anti-COX-2 antibody (1∶1000; Cayman Chemical), mouse anti-GFAP antibody (1∶1000; Clone G-A-5, Sigma-Aldrich), rabbit anti-vWF antibody (1∶500; Abcam, Cambridge, UK), and mouse anti-NeuN antibody (1∶1000; Millipore, Billerica, MA)] in blocking solution for overnight. After incubation, the sections were washed with PB, and incubated with biotin-conjugated secondary antibodies [anti-mouse IgG (1∶500; Vector Laboratories, Burlingame, CA) for TTF-1 (NeoMarkers), NeuN, and GFAP and anti-rabbit IgG (1∶500; Vector Laboratories) for TTF-1 (Santa Cruz Biotechnology), COX-2, and vWF] in blocking solution for 2 h. After being washed with PB, the sections were incubated with avidin-biotinylated HRP-complex (ABC, Vector Laboratories) for 2 h and then reacted with Tyramide Signal Amplification system (NEN Life Science, Boston, MA). For double immunofluorescence detection, after generation of the first signals, sections were incubated in 0.3% H_2_O_2_ for 30 min followed by three 10-min washes in PB, and then the second signals were developed. After processing, the sections were mounted with a cover slip and photographed using fluorescence microscopy.

### Cell culture and assays for luciferase activity

Rat neuroblastoma B35 cells and rat glioma C6 cells were grown in DMEM supplemented with high glucose (4.5 g/L) and 10% fetal bovine serum at 37°C in a humidified atmosphere with 5% CO2. Twenty-four h after seeding the cells in 12-well plates, they were transiently transfected with the rat COX-2 promoter-luciferase reporter construct (COX-2-P) using Lipofectamine/PLUS (Invitrogen Life Technologies, Gaithersburg, MD) along with different concentrations of the expression vector pcDNA 3.1-zeo (Invitrogen) containing the rat TTF-1 coding region (TTF-1-pcDNA). Transfection efficiency was normalized by co-transfecting the β-galactosidase reporter plasmid (pCMV-β-gal; Clontech, Palo Alto, CA) at 20 ng/well. The transfected cells were harvested 24 h after transfection and used for luciferase and β-galactosidase assays, as previously reported (20).

### PGE2 measurement

Fifty microliters of collected medium from cultured C6 and B35 cells were used for analysis of PGE2 using a PGE2 EIA kit (Cayman Chemicals) following the manufacturer's instructions. Both the samples and standards were assayed in parallel.

### EMSAs

Expression and purification of the TTF-1 HD have already been described [43]. Double-stranded oligodeoxynucleotides, labelled at the 5′ end terminal with ^32^P, were used as probes in the gel-retardation assays. Sequences of used oligonucleotides are shown in Fig. S2 and Table 1. The oligonucleotides C and Cβ were used as positive and negative control, respectively [44]. The gel-retardation assay was performed by incubating protein and DNA in a buffer containing 20 mM Tris-HCl (pH 7.6), 75 mM KCl, 0.25 mg/ml BSA, 5 mM DTT, 50 g/ml calf thymus DNA, 10% glycerol for 30 min at room temperature. TTF-1HD was used at 150 nM. Oligonucleotides were used at the concentration of 5 mM. Protein-bound DNA and free DNA were separated on native 7.5% polyacrylamide gel run in 0.5x TBE (1x TBE = 45 mM Tris/borate/1 mM EDTA), for 1.5 h at 4°C. Gels were fixed and exposed to phosphoimager (GS525; Bio-Rad, Hercules, CA). Signals corresponding to protein-bound and free DNA were quantified by using the Multi-analyst software. Binding of TTF-1HD to oligonucleotides of the COX-2 promoter was expressed as a percentage of the TTF-1HD binding to the C oligonucleotide. In competition experiments, cold oligonucleotides were used at 5 and 20 fold excess of the labelled probe. To confirm the presence of immunoreactive TTF-1 in nuclear extracts, proteins were incubated with 3 µl of undiluted TTF-1 antibody (NeoMarkers) for 30 min at room temperature before performing binding reactions.

### ChIP assay

After lysis of the C6 cells, nuclei were extracted and resuspended with nuclear lysis buffer (50 mM Tris, pH 8.1, 10 mM EDTA, 1% SDS, and protease inhibitors). Chromatin was sheared by sonication and diluted 5 fold in ChIP dilution buffer (0.01% SDS, 1.1% Triton X-100, 1.2 mM EDTA, 16.7 mM Tris, pH 8.1, 167 mM NaCl, and protease inhibitors). The reactions were incubated with 1 µg of antibodies against TTF-1 (Santa Cruz Biotechnology) at 4°C for overnight. Immune complexes were collected by reacting with 60 µl of the salmon sperm DNA/protein A agarose for 1 h at 4°C, and then washed consecutively for 5 min each with buffers (0.1% SDS, 1% Triton X-100, 2 mM EDTA, 20 mM Tris, pH 8.1) containing different concentration of salts (150 mM–500 mM), and 0.25 M LiCl. DNA from the protein-DNA cross-links was extracted by incubating the reactions with solution (1% SDS, 0.1 M NaHCO_3_, 10 µg RNase, and 0.3 M NaCl) at 65°C for 4 h and was further purified with phenol/chloroform. PCR amplification was performed using 35 cycles of 94°C for 30 sec, 54°C for 30 sec and 72°C for 30 sec, proceeded by 94°C for 5 min, and followed by 72°C for 10 min. Sequence information about PCR primer sets for ChIP assays are presented in Information S1.

### Icv administration of AS TTF-1 ODN

To determine effect of blocking TTF-1 expression on COX-2 synthesis, a phosphorothioate AS TTF-1 ODN (GenoTech Corp., Daejeon, Korea) was delivered into the lateral ventricle (coordinates: AP = 1.0 mm caudal to the bregma; V = 3.6 mm from the dura mater; L = 0.16 mm from the midline) of adult male rats. The AS TTF-1 ODN used to disrupt TTF-1 synthesis (5′-GAC TCA TCG ACA TGA TTC GGC GTC-3′) was directed against the sequence surrounding the first ATG codon of TTF-1 mRNA as previously reported (20). As a control, a scrambled sequence of identical base composition was used (5′-AGT CCT ACT CGG TAC GTA TGC AGC-3′). For the icv injection, the ODNs were diluted to a final concentration of 0.5 nmol/ µl of artificial cerebrospinal fluid [20], and injected into the lateral ventricle using an infusion syringe pump (KDS 100; KD scientific, Holliston, MA). The animals were euthanized 24 h after ODNs injection, and brain tissues were prepared.

### Measurements of body temperature

Abdominal temperature was measured in male Sprague-Dawley rats using biotelemetry transmitters (Mini-Mitter, Bend, OR) implanted into the abdominal cavity. Prior to surgery, rats were anesthetized with tribromoethanol (250 mg/kg B.W., Sigma-Aldrich). After a week of recovery, AS TTF-1 ODN and its SCR ODN (2 nmol /4 µl, respectively) were injected with an infusion syringe pump. After injection of ODNs, temperature was recorded on top of the receivers (model RA 1000; Mini-Mitter). A data acquisition system (Vital View; Mini-Mitter) was used for automatic control of data collection and analysis. Body temperature was recorded at 10-min intervals for 24 h after the injection of ODNs.

### Statistics

Student's *t*-test was used for comparison of two groups. Differences among more than three groups were analyzed by one-way ANOVA with Dunnett's multiple comparison post-hoc tests.

## Supporting Information

Figure S1 **Localization of COX-2 protein in several different structures of the rat brain.** The SCR ODN (A–C) or TTF-1 AS ODN (D–H) was injected into the lateral ventricle of 2-month-old male rats. Brain sections containing the cerebral cortex (A, D, G), hippocampus (B, E, H) and piriform cortex (C, F) were incubated with COX-2 antibody alone (green, A–F) or together with NeuN antibody (red, G, H). Only blood vessel-like cells (closed arrow heads on D, E) revealed a clear change in COX-2-immunoreactivity by the AS ODN. Double immunohistochemistry revealed that these blood vessel-like cells are absent of NeuN-immunoreactivity (closed arrow heads on G, H). Open arrow heads indicating representative cells co-expressing COX-2- and NeuN-immunoreactivities (G, H). Scale bar = 100 µm.
(TIF)

Figure S2 **Nucleotide sequence of 5′-flanking region of the rat COX-2 gene.** To find possible TTF-1 binding motifs, DNA sequences for the rat COX-2 gene (NCBI GenBank database, accession No. L11611) were analyzed. Nucleotides are numbered by assigning position +1 to the transcriptional start site (indicated with arrow). Position of putative TTF-1 binding motifs, 5′-CAAG-3′ and 5′-CTTG-3′, are indicated (red and underlined). Several transcription factor binding sites and TATA box, based on sequence analysis, are also indicated (underline). NF-κB, nuclear factor kappa B; C/EBPβ, CCAAT/enhancer-binding protein beta; CRE, cAMP response element.
(TIF)

Information S1 **Primer sets for ChIP assays.** The following primer sequences were used for PCR amplification of the indicated TTF-1 binding domains.
(DOCX)
