# Supplementary material for: TTF-1 Action on the Transcriptional Regulation of Cyclooxygenase-2 Gene in the Rat Brain
Source: PLoS One. 2011 Dec 13;6(12):e28959. doi: 10.1371/journal.pone.0028959 (PMC3236776; doi:10.1371/journal.pone.0028959)
Supplement: Information S1 — Primer sets for ChIP assays. The following primer sequences were used for PCR amplification of the indicated TTF-1 binding domains. (DOCX) [file pone.0028959.s003.docx]

**Supplementary information S3.** Primer sets for ChIP assays. The following primer sequences were used for PCR amplification of the indicated TTF-1 binding domains.

| TTF-1 binding domains | Sense primer | Antisense primer |
| --- | --- | --- |
| -2624 | 5’-CCA AAA TTT ACC TAA CCA TCT ACC-3’ | 5’-AGG AAA CGC ATT AAC ATC TTC G-3’ |
| -2540 | 5’-TGC GTT TCC TCA TTT TCC TTT-3’ | 5’-GCG CGA TGA TAA AGA TGC TC-3’ |
| -2361 | 5’-TGT ATT TTG TAC ACA GCA GGC AC-3’ | 5’-GGA ATA TTA ATT GTC CCA AAC TAA-3’ |
| -2331 | 5’-CAA TTA ATA TTC CCT TGT CAT CAG C-3’ | 5’-AGC AAT TTT GAA AGG CAG TCC-3’ |
| -2203 ~ -2170 | 5’-GCC GAC ATA CTG TGC TCT GG-3’ | 5’-TGC ATG GTT CAG TTG GTT GA-3’ |
| -2039 | 5’-TAG GGA GCC CTC TTC ACC TC-3’ | 5’-GGG AAT AAT TAC CTT CAG ACT CCT T-3’ |
| -2008 | 5’-GGT AAT TAT TCC CTA TGC CTT GC-3’ | 5’-TCT CAG GAG AGC CAG CTG TA-3’ |
| -1900 | 5’-CCT CGT TTC TTT GAA TTC CC-3’ | 5’-GGT CGG AAA TGG GTG GTA GT-3’ |
| -1662, -1657 | 5’-ACT ACC ACC CAT TTC CGA CC-3’ | 5’-AGT GAA TTG CAT GGG CTC AA-3’ |
| -1549 | 5’-TTT ACA TGG CTC CTA GCC GA-3’ | 5’-AAT CAT TCC GTG TAT GGC ACT C-3’ |
| -1421 | 5’-GGG AGT GCC ATA CAC GGA AT-3’ | 5’-GAG AAA CAG CCC TTC GGA AA-3’ |
| -1075 | 5’-TTT TGT TTT GCT CTG GTT TGT T-3’ | 5’-AAA ATG AGG AAA CTA AAA CAT TCA-3’ |
| -1032 | 5’-CGT ACG GTT TAA TTG AAT GTT TT-3’ | 5’-CCC ACT GTG TTT GGT GTT GG-3’ |
| -911 | 5’-TTC CAT CCT CAG ATC CTC CC-3’ | 5’-TTT TCC CGA TAA CTG GCC TT-3’ |
| -805 | 5’-AGG CCA GTT ATC GGG AAA AA-3’ | 5’-AAG GTA TTT TCC TCC CTG CTG-3’ |
| -592 | 5’-TGC GGT GGA CAC TTA GCA TT-3’ | 5’-GCT GTC AGG GTG ACA TCT GC-3’ |
| -441 | 5’-TAG GAC TGC GGA GCC TGG-3’ | 5’-CAC TGG GGC GCA GTC TGA-3’ |
| -407 | 5’-CAC GTC AGA CTG CGC CCC-3’ | 5’-AAT GTC ACA GCT TCC CTC CC-3’ |
| -351 | 5’-GGG AGG GAA GCT GTG ACA TT-3’ | 5’-GCT CTC TTT GAG GTC TCG GG-3’ |
| -284 | 5’-AGG AAA CCC GAG ACC TCA AA-3’ | 5’-AGA GCT GCA CCG CCC TCT-3’ |
| -223 | 5’-TGG AAG GAT GCA GAG GGC-3’ | 5’-CTC CGC TTC CAA TTG CAT AA-3’ |
| -23 | 5’-TTA TGC AAT TGG AAG CGG AG-3’ | 5’-GTT TGA CAA CTG GCC GCT AA-3’ |
